# Changes in the Incidence of Cardiovascular Diseases during the COVID-19 Pandemic in Korea

**DOI:** 10.3390/jpm12071183

**Published:** 2022-07-20

**Authors:** Hyo Geun Choi, Dae Myoung Yoo, Yoo Hwan Kim, Mi Jung Kwon, Joo-Hee Kim, Joon Ho Song, Ji Hee Kim

**Affiliations:** 1 Hallym Data Science Laboratory, Hallym University College of Medicine, Anyang 14068, Korea; pupen@naver.com (H.G.C.); ydm1285@naver.com (D.M.Y.); 2 Department of Otorhinolaryngology-Head & Neck Surgery, Hallym University College of Medicine, Anyang 14068, Korea; 3 Department of Neurology, Hallym University College of Medicine, Anyang 14068, Korea; drneuroneo@gmail.com; 4 Department of Pathology, Hallym University College of Medicine, Anyang 14068, Korea; mulank@hanmail.net; 5 Division of Pulmonary, Allergy, and Critical Care Medicine, Department of Medicine, Hallym University College of Medicine, Anyang 14068, Korea; luxjhee@gmail.com; 6 Department of Neurosurgery, Hallym University College of Medicine, Anyang 14068, Korea; song@hallym.or.kr

**Keywords:** cardiovascular diseases, cerebrovascular disease, coronavirus disease, COVID-19, heart disease, incidence, stroke

## Abstract

There is scarcity in the evidence addressing the indirect impact of the COVID-19 pandemic on the epidemiology of CVDs. In this study we aimed to examine possible changes in the incidence of CVDs in Korea during the COVID-19 pandemic. ICD-10 codes of six common CVDs (cerebral hemorrhage, cerebral infarction, myocardial infarction, ischemic heart disease, cardiac failure, and arrhythmia) were collected from clinical visits between January 2018 and March 2021 using the National Health Insurance service database, which stores data on all citizens of Korea (~50 million people). The number and distribution of monthly visits for CVDs were compared before and during the COVID-19 pandemic, and the differences were analyzed using the Mann–Whitney U test and Levene’s test. Our data showed similar incidences of cerebral hemorrhage and ischemic heart disease, a lower incidence of cerebral infarction, and higher incidences of myocardial infarction, cardiac failure, and arrhythmia during COVID-19. Despite statistical differences, the changes in incidences were not considered meaningful. The monthly incidences also remained similar throughout the year, without seasonal variations, both before and during the COVID-19 outbreak. This study found no significant changes in the incidences or monthly variation in CVDs due to the COVID-19 pandemic in Korea.

## 1. Introduction

Since the first case of severe acute respiratory syndrome coronavirus-2 (SARS-CoV-2) infection that was reported in December 2019, and until 9 February 2022, the COVID-19 pandemic has affected over 400 million people worldwide, with more than 5.7 million fatalities [1]. The clinical presentation of COVID-19 varies considerably in terms of symptoms, severity, and organ involvement, ranging from asymptomatic to multi-organ failure. COVID-19 emerged as a disease that not only caused substantial clinical manifestations related to the infection itself but also contributed to indirect consequences on various other diseases, the so-called collateral damage. These consequences might not be only related to the pathophysiologic changes caused by the infection itself, but also to other social and environmental changes triggered by the COVID-19 disease and pandemic.

Cardiovascular disease (CVD) is a general term that refers to conditions that affect the cardiovascular system, which consists of the heart and blood vessels, such as coronary artery disease, cerebrovascular disease, peripheral artery disease, and aortic atherosclerosis [2]. Coronary artery disease, also known as coronary heart disease, is caused by decreased myocardial perfusion that causes angina, myocardial infarction (MI), and/or heart failure, and accounts for one-third to one-half of all CVDs. Cerebrovascular disease refers to conditions that typically present with neurological deficits of sudden onset, such as strokes, also known as cerebrovascular accidents, and transient ischemic attacks. Altogether, CVDs are the leading cause of death and major contributors to disability worldwide, affecting people of all ages, ethnicities, and socioeconomic levels [3].

It has been established that CVDs are caused by a set of chronic conditions that arise from a complex interaction between genetic predisposition and environmental influences, resulting in progressive deterioration in the structure and the function of cardiovascular tissues [4]. In particular, changes in the environment due to migration to different geographic locations, modifications in lifestyle choices, and shifts in social policies and cultural practices are increasingly being recognized as modifiable risk factors for CVDs [5]. Consequently, given the overwhelming impact of the COVID-19 pandemic on the lifestyles and environments of individuals and on societies at a larger level, this pandemic might contribute to a substantial change in the incidence of CVDs. For example, prioritizing the treatment of COVID-19 and concentrating the efforts of healthcare facilities in this direction could result in a reduction in the treatment of other chronic conditions or the detection of other new-onset diseases not related to COVID-19, such as CVDs. In addition to that, the alterations in lifestyles forced by COVID-19 and the consequences of this disease on the mental health of individuals can also lead to an increased risk of CVDs development. This might be driven by the policy of social distancing forced by COVID-19 to minimize the risk of contagion, which may endorse sedentary behavior and unhealthy eating habits, as well as stress, loneliness, and anxiety due to social isolation, which all could have detrimental effects driving the development of CVDs.

Currently, the majority of reported studies on COVID-19 and CVDs have mainly focused on the direct effects of SARS-CoV-2 on the cardiovascular systems or the impacts of underlying CVDs on COVID-19 incidence and subsequent morbidity and mortality [6,7]. However, there is scarcity in the evidence addressing the indirect impact of the COVID-19 pandemic on the epidemiology of CVDs in both Korea and the world. Therefore, in this study, we aimed to examine whether there are changes in the incidence of CVDs in Korea associated with the social and environmental influences of the COVID-19 pandemic through a large nationwide study. To achieve this aim, we investigated and compared the number of diagnostic registrations for CVDs, including strokes, MI, ischemic heart disease, cardiac failure, and arrythmia, throughout the Korean population before and after the outbreak of COVID-19.

## 2. Materials and Methods

### 2.1. Ethics

The ethics committee of Hallym University (2021-11-004) approved the use of the data needed to be collected by this study. This study was approved by the Institutional Review Board and exempted from the need for written informed.

### 2.2. EthicsParticipants and Measurement

The entire Korean population is obligated to register with the National Health Insurance service (NHI). To collect data used for this study, we used records from the National Health Insurance service database, which thus included all citizens of Korea (approximately 50 million people) without exception. NHI contains all public and private information on patient demographics (age, sex at entry, income, and residency), medical use/transaction information, and claim database (diagnosis/prescriptions/consultation statements). Therefore, it can be used as a population-based, nationwide study for various diseases. The data gathered included diagnostic codes of CVDs for all Korean people across the full range of healthcare settings, from the primary clinic to the tertiary hospital, regardless of geographic locations. To determine the changes in the incidence of CVDs before and after the COVID-19 outbreak, clinic visits for CVDs were captured using diagnostic codes related to these diseases from January 2018 to May 2021. The first COVID-19 patient in Korea was identified on 20 January 2020, and disease prevention and control began in earnest in Korea in March 2020. Accordingly, the period ‘before the COVID-19 pandemic’, or also called ‘comparison period’, was designated as until February 2020 and ‘during the COVID-19 pandemic’ was set as March 2020 and onwards.

Information on six CVDs, which are the most common in primary clinics, was extracted from the database to assess the monthly incidence of each CVD. Each disease was identified using the diagnostic codes of the International Classification of Diseases-10 (ICD-10) as follows: cerebral hemorrhage (I60, I61, I62), cerebral infarction (I63), myocardial infarction (I21, I22), ischemic heart disease (I20, I21, I22, I23, I24, I25), cardiac failure (I50), and arrhythmia (I48, I49). The data used in this study included the medical records of entire hospitals or clinics and matched the diagnoses of patients with their own residential registration numbers; it was thus possible to estimate the number of diagnostic registrations without duplication or overlap.

### 2.3. Statistics

The difference in the mean number of diagnostic registrations before and during the COVID-19 pandemic was compared using the Mann–Whitney U test for nonparametric values. The difference in the distribution of monthly diagnostic registrations before and during the COVID-19 pandemic was compared using Levene’s test for nonparametric values [8]. All results were stratified by age (0–19 years old, 20–59 years old, and 60+ years) and sex in sub-group analyses. 

Two-tailed analyses were conducted, and the level of statistical significance was set at *p* values < 0.05 for all analyses. All statistical analyses were performed using SPSS version 22.0 (IBM, Armonk, NY, USA).

## 3. Results

From January 2018 to May 2021, cerebral hemorrhage and cerebral infarction were recorded in 1,327,131 and 6,795,513, respectively. A total of 1,243,740 and 10,554,338 were diagnosed with myocardial infarction and ischemic heart disease. In addition, 1,471,549 and 3,683,612 cases were registered with cardiac failure and arrhythmia.

**Figure 1:**
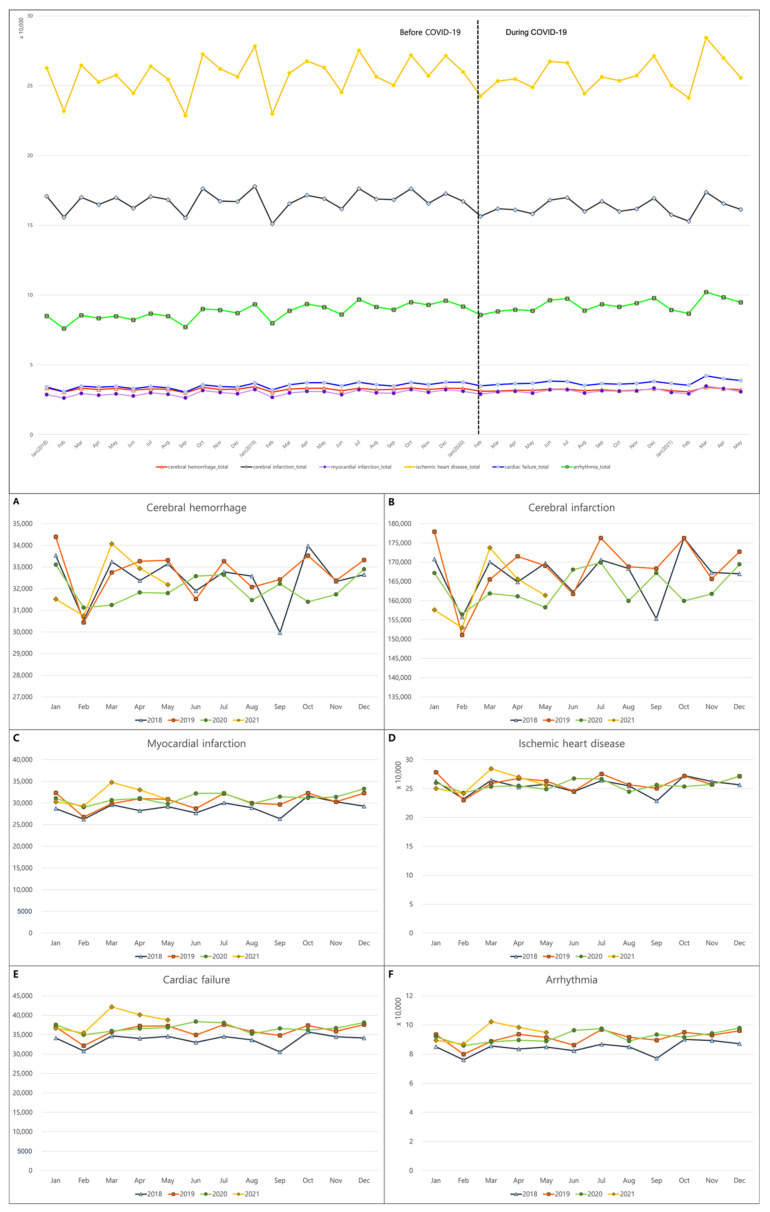
Monthly incidence of cardiovascular diseases during 2018, 2019, 2020, and 2021. The incidences of (**A**) cerebral hemorrhage; (**B**) cerebral infarction; (**C**) myocardial infarction; (**D**) ischemic heart disease; (**E**) cardiac failure; and (**F**) arrhythmia.

The nationwide data showed a similar incidence of cerebral hemorrhage and ischemic heart disease before and during the COVID-19 pandemic. It, however, revealed a slightly reduced registration of cerebral infarction diagnosis (*p* = 0.045) and an increased number of diagnoses for MI, cardiac failure, and arrhythmia during the COVID-19 pandemic (*p* = 0.006, 0.001, and 0.005, respectively; Table 1 and Figure 1).

Although fewer people seemed to have been diagnosed with cerebrovascular accidents, such as cerebral hemorrhage and cerebral infarction, in February and September, no clear seasonality of cerebrovascular accidents was found before the COVID-19 pandemic, and this remained unchanged during the COVID-19 pandemic (Figure 1). Similarly, concerning the rest of the CVDs, few variations in the number of incidences per month were also observed before and during the COVID-19 pandemic. In other words, individuals with MI, ischemic heart disease, cardiac failure, and arrhythmia also displayed a similar number of diagnoses throughout the year, regardless of the COVID-19 pandemic (Figure 1).

A subgroup analysis based on sex showed that the incidence of either cerebral infarction or ischemic heart disease in men was similar before and during the COVID-19 pandemic. However, the diagnosis of cerebral hemorrhage in men was reported to be somewhat decreased during the COVID-19 pandemic (*p* = 0.019), whereas there was an increased incidence of MI, cardiac failure, and arrhythmia in men during the COVID-19 pandemic (*p* = 0.001, *p* <0.001, and *p* = 0.002, respectively; Table 2). Meanwhile, a subgroup analysis of women showed a slightly lower number of diagnostic registrations for all CVDs except for cardiac failure and arrythmia during the COVID-19 pandemic compared to that in the period before. However, these results for cerebral hemorrhage and MI were not statistically significant (Table 2).

With regard to the subgroups according to the age, in individuals aged < 19 years, the number of diagnostic registrations for all CVDs except for cerebral infarction was lower during the COVID-19 pandemic than during the comparison period (*p* < 0.05 for all, Table 3). Compared to those before the COVID-19, the incidences for cerebral hemorrhage, cerebral infarction, and ischemic heart disease also decreased in the 20–59-year-old subgroup during the COVID-19 pandemic (*p* < 0.001 for all, Table 3). The results of the subgroup aged > 60 years showed that the number of people diagnosed with MI, cardiac failure, and arrhythmia increased during the COVID-19 pandemic (*p* < 0.001 for all, Table 3).

## 4. Discussion

Overall, our findings demonstrated that the trends in the incidence of CVDs in Korea during the COVID-19 pandemic are comparable to those before the pandemic. Furthermore, there was no significant variation in the monthly distribution of the incidences of CVDs before and after the COVID-19 outbreak.

The results of our study were not in accordance with the results of research studies from other countries exploring the effect of the COVID-19 pandemic on CVDs. A US study reported that emergency department visits in a 4-week period during COVID-19 pandemic were significantly lower than those in the same 4-week period in the previous year when the COVID-19 pandemic had not started [9]. This study showed that the number of visits for conditions, including nonspecific chest pain and acute MI, decreased during the COVID-19 pandemic, implying that some persons could be having delayed care for such serious conditions that might lead to an increased mortality. A preliminary analysis of another study during the early phase of the COVID-19 pandemic provided an estimate of a 38% reduction in ST-segment elevation MI activations in catheterization laboratories in nine high-volume centers in the US [10].

In addition to the US, soon after the WHO declared COVID-19 as a global pandemic, anecdotal evidence and surveys from Europe indicated a decline in the number of patients presenting with CVDs requiring emergency procedures [11]. A study from Venice showed a reduction in calls for chest pain without ST-segment elevation MI (−54%, *p* = 0.024) and out-of-hospital cardiac arrests with resuscitation efforts (−38%, *p* = 0.04). Likewise, data from 73 healthcare system in Spain disclosed treating 40% fewer patients with ST-segment elevation MI during the COVID-19 pandemic (from 16 March 2020 to 22 March 2020) and explained that a significant number of patients with MI did not seek medical attention because they were afraid of being infected at the hospitals [12]. A recent systematic review identified that 11 studies revealed a decline in admissions for acute coronary syndrome, ranging from −40% to −50%, and 5 studies showed a reduction in stroke admissions of 12% to 40% when comparing the COVID-19 period to non-COVID-19 periods [13].

Unlike other countries, the reason why the incidence of CVDs in Korea was maintained without significant changes is uncertain; however, several explanations could possibly justify this. First, the burden that COVID-19 placed on the healthcare system in Korea was lower than that in some other countries. Up until 9 May 2020, the cumulative number of COVID-19 reported cases and deaths in Korea was 10,840 and 256, respectively [14], which is less than 1% of all citizens. A survey in the UK, where the number of confirmed cases soared in the early stages of COVID-19, revealed that fear of being exposed to COVID-19 is the most common reason for the decrease in admission for acute coronary syndrome, followed by worries of putting pressure on an already overburdened healthcare system [15]. Additionally, several reports have observed a shortening of the length of hospital stays and lengthening of symptom-to-door times due to capacity issues during the pandemic compared to those in pre-pandemic periods [16]. However, during the extensive outbreak of COVID-19 in Korea, various strategies were adopted to mitigate community transmission and prevent nosocomial infections in hospitals, such as the establishment of dedicated COVID-19 hospitals. The measures used during the Middle East respiratory syndrome (MERS) outbreak were critical to effectively address the crisis of COVID-19, given the shortage in healthcare resources [17]. Combining the aforementioned findings, it seems that the resources and efficacy of healthcare systems in Korea were relatively well sustained during the pandemic; as a result, there were no major restrictions on the use of appropriate medical services by the public for non-COVID-19-related conditions such as CVDs.

Second, reports from other countries disclosed that people might be reluctant to seek hospital care for fear of infection or contagion, which substantially threatened the accessibility of the diagnostic and therapeutic management of diseases [18,19]. However, a Korean survey showed that the health status and behavior of the majority of Koreans were not heavily affected by the COVID-19 outbreak. In addition, whereas a cross-sectional survey of other countries identified that people who reported symptoms of stress, anxiety, and depression were more likely to avoid presentation to medical attention despite the need [19], Korean studies indicated that the mental health symptoms of a number of Korean people were not significantly increased during the COVID-19 pandemic [20,21]. In this context, the medical visits for CVDs in Korea were not significantly influenced by COVID-19, which can be attributed to the lack of psychological distress during the pandemic period.

Third, the COVID-19 lockdown implemented by the governments of various countries to further suppress the spread of this virus might have impacted the lifestyle of people and made them more prone to habits that increase the risk of CVDs. For example, owing to the COVID-19 lockdown, there have been increases in unhealthy eating and alcohol consumption resulting from stress and social isolation, and a decrease in physical activity with an increase in sedentary lifestyles. These modifiable risk factors might thus contribute to the development of CVDs [22]. Due to the fact that the COVID-19 pandemic period investigated in this study was relatively short, the effect on CVD development might not have been large; thus, there appeared to be no significant change in the incidence of CVDs.

A striking finding of our study was that there were few variations in the number of CVD incidences per month before and during the COVID-19 pandemic. Seasonality generally refers to the ‘winter peaks’ in CVD-related hospitalizations and mortality, where the rates in winter are typically 10–20% higher than those during ‘summer troughs’. Several reports have showed an increase in the incidence of stroke in response to dramatic temperature changes in spring and autumn. Unexpectedly, our data showed little variation in the incidence per month of all CVDs both before and during the COVID-19 pandemic. This might be because seasonal variation in incidence is probably caused by a wide range of environmental factors, including ambient temperature or rapid temperature changes, and complex interactions between these environmental factors and the susceptibility of individuals can ultimately affect disease incidence.

Our findings should, however, be considered with some cautions: First, our data included all patients with corresponding diagnostic codes for CVDs, which does not include patients with new occurrence of CVDs but only those utilizing medical facilities for CVDs-related complaints. Although the incidence in newly diagnosed patients with CVD was not accurately established in this study, we tried to indirectly assess the incidence using the number of medical visits for CVD as a surrogate marker. Additionally, it was possible to obtain monthly medical visits for CVDs and the changes in visit patterns or trends before and after COVID-19 because this investigation was conducted monthly. Second, the possibility that patients with CVDs included in this study had concomitant confirmed COVID-19 cannot be ruled out. This is important because cardiovascular comorbidities are common in patients with COVID-19, and COVID-19 patients can also show a variety of cardiac presentations [23], which can be a confounding factor in our results. In fact, the cumulative number of COVID-19-identified cases in Korea was 140,337 by 31 May 2021, and so this possibility cannot be completely excluded. Subsequently, the effect of COVID-19 on the incidence of CVDs during the investigation period of this study is likely to be both direct and indirect. Third, the possibility that the incidence of CVDs was diagnosed lower than the actual incidence during the period cannot be completely excluded due to the prioritization of COVID-19 care and the resulting burden on the healthcare system. Fourth, we only investigated the short-term effects of the pandemic on medical visits for CVDs and were not able to assess or predict the long-term consequences. Finally, we did not investigate the number of diagnoses based on the healthcare setting, such as whether CVD diagnoses were registered at a primary clinic or a tertiary hospital. Thus, we could not determine how the healthcare environment affected the medical visits for CVDs during the COVID-19 pandemic. Further research is thus warranted to clarify many of the unanswered questions. Moreover, further analyses for the COVID-19-related heart diseases, such as myocarditis and pericarditis, are required to comprehend both the direct and indirect impacts of COVID-19 on the various CVDs as well.

## 5. Conclusions

In conclusion, understanding the indirect influences of the pandemic on the incidence of CVDs is imperative to plan for recovery, adopt the most efficient responses to this ongoing disaster, and guide the response to future health crisis. Although the incidence of CVDs in Korea has not changed significantly before and after COVID-19, it is essential to continuously maintain patient awareness with respect to acute CVDs such as MI and stroke, which could be more fatal than COVID-19. It is also important to plan public health measures to ensure that, during pandemics and crises, the burden on the healthcare system does not hinder the diagnosis and treatment of critical diseases such as CVDs and ensure that the measures taken to mitigate the spread of the pandemic does not indirectly increase the risk of developing some diseases, such as CVDs.

## Figures and Tables

**Table 1:** Mean and standard deviation of the monthly incidence in six cardiovascular diseases before and during the COVID-19 pandemic.

Cardiovascular Diseases	Before COVID-19		During COVID-19		*p*-Value of Difference	
	Mean	Standard Deviation	Mean	Standard Deviation	Mean	Variance ^2^
Cerebral hemorrhage	32,534.9	1091.3	32,081.5	839.7	0.062	0.327
Cerebral infarction	167,184.9	6844.2	163,247.1	5561.7	0.045 ^1^	0.483
Myocardial infarction	29,710.4	1764.7	31,417.9	1479.6	0.006 ^1^	0.182
Ischemic heart disease	256,915.2	13,436.4	258,302.9	11,453.4	0.725	0.837
Cardiac failure	34,997.2	1959.6	37,441.5	1866.2	0.001 ^1^	0.197
Arrhythmia	87,901.8	5475.0	93,210.9	4580.0	0.005 ^1^	0.374

^1^ Mann–Whitney U test, significance at < 0.05; ^2^ Levene’s test in non-parametric data.

**Table 2:** Mean and standard deviation of the monthly incidence in six cardiovascular diseases before and during the COVID-19 pandemic stratified by sex in subgroup analysis.

Cardiovascular Diseases	Before COVID-19		During COVID-19		*p*-Value of Difference	
	Mean	Standard Deviation	Mean	Standard Deviation	Mean	Variance ^2^
Men						
Cerebral hemorrhage	15,820.1	554.2	15,428.9	392.8	0.019 ^1^	0.157
Cerebral infarction	91,761.8	3982.4	91,122.3	3254.4	0.482	0.687
Myocardial infarction	22,869.3	1422.4	24,576.5	1209.9	0.001 ^1^	0.194
Ischemic heart disease	159,339.0	8617.7	163,613.5	7348.6	0.244	0.519
Cardiac failure	13,698.4	860.6	15,034.9	741.7	<0.001 ^1^	0.272
Arrhythmia	48,138.0	3011.7	51,459.4	2467.8	0.002 ^1^	0.376
Women						
Cerebral hemorrhage	16,714.8	546.0	16,652.6	460.5	0.417	0.970
Cerebral infarction	75,423.1	2928.7	72,124.7	2340.8	0.001 ^1^	0.150
Myocardial infarction	7301.2	371.7	7232.3	290.8	0.372	0.410
Ischemic heart disease	97,576.2	5083.4	94,689.4	4173.8	0.048 ^1^	0.337
Cardiac failure	21,298.8	1110.9	22,406.6	1134.9	0.004 ^1^	0.468
Arrhythmia	39,763.9	2476.2	41,751.5	2143.9	0.020 ^1^	0.413

^1^ Mann–Whitney U test, significance at <0.05; ^2^ Levene’s test in non-parametric data.

**Table 3:** Mean and standard deviation of the monthly incidence in six cardiovascular diseases before and during the COVID-19 pandemic stratified by sex in subgroup analysis.

Cardiovascular Diseases	Before COVID-19		During COVID-19		*p*-Value of Difference	
	Mean	Standard Deviation	Mean	Standard Deviation	Mean	Variance ^2^
Age 0–19 years old						
Cerebral hemorrhage	229.8	15.3	209.1	20.1	0.001 ^1^	0.699
Cerebral infarction	140.5	10.9	140.6	19.1	0.516	0.356
Myocardial infarction	5.7	2.8	2.9	1.0	0.001 ^1^	0.059
Ischemic heart disease	141.6	15.8	112.5	13.8	<0.001 ^1^	0.230
Cardiac failure	38.5	5.1	32.7	6.4	0.004 ^1^	0.958
Arrhythmia	730.3	98.1	627.4	92.2	0.008 ^1^	0.879
Age 20–59 years old						
Cerebral hemorrhage	11,956.6	486.0	11,056.7	300.7	<0.001 ^1^	0.146
Cerebral infarction	27,690.0	1200.3	25,649.7	918.1	<0.001 ^1^	0.124
Myocardial infarction	10,097.7	535.8	10,152.9	458.5	0.914	0.690
Ischemic heart disease	62,596.5	3410.9	57,833.3	2334.2	<0.001 ^1^	0.105
Cardiac failure	5050.0	261.5	5171.9	203.9	0.218	0.367
Arrhythmia	22,121.1	1108.3	21,512.7	924.4	0.070	0.599
Age 60+ years old						
Cerebral hemorrhage	20,370.5	710.6	20,836.4	616.0	0.099	0.454
Cerebral infarction	139,430.9	5732.9	137,520.0	4734.1	0.185	0.909
Myocardial infarction	19,617.3	1256.5	21,270.7	1075.3	0.000 ^1^	0.136
Ischemic heart disease	194,264.8	10,573.5	200,438.3	9495.0	0.130	0.780
Cardiac failure	29,922.1	1728.0	32,250.5	1686.3	0.001 ^1^	0.197
Arrhythmia	65,089.5	4497.1	71,109.1	3680.5	0.000 ^1^	0.330

^1^ Mann–Whitney U test, significance at <0.05; ^2^ Levene’s test in non-parametric data.

## Data Availability

Restrictions apply to the availability of these data. Data were obtained from the Korean National Health Insurance Sharing Service (NHISS) and are available at https://nhiss.nhis.or.kr (accessed on 10 January 2022) with the permission of the NHISS.
